# Using Life History Calendars to Estimate in Utero and Early Life Pesticide Exposure of Latinx Children in Farmworker Families

**DOI:** 10.3390/ijerph17103478

**Published:** 2020-05-16

**Authors:** Sara A. Quandt, Dana C. Mora, Theresa L. Seering, Haiying Chen, Thomas A. Arcury, Paul J. Laurienti

**Affiliations:** 1 Department of Epidemiology and Prevention, Division of Public Health Sciences, Wake Forest School of Medicine, Winston-Salem, NC 27157, USA; 2 Department of Family and Community Medicine, Wake Forest School of Medicine, Winston-Salem, NC 27157, USA; dmora@wakehealth.edu (D.C.M.); tarcury@wakehealth.edu (T.A.A.); 3 Department of Anthropology, Lawrence University, Appleton, WI 54911, USA; Theresa.l.seering@lawrence.edu; 4 Department of Biostatistics and Data Science, Division of Public Health Sciences, Wake Forest School of Medicine, Winston-Salem, NC 27157, USA; hchen@wakehealth.edu; 5 Department of Radiology, Wake Forest School of Medicine, Winston-Salem, NC 27157, USA; plaurien@wakehealth.edu

**Keywords:** Latino/Hispanic, agricultural health, health disparities, migrant and seasonal farmworkers, exposure assessment

## Abstract

(1) Background: Early life exposure to neurotoxic chemicals can have later impacts on child health. Most research designs must assume that current exposure is similar to past. Life history calendar methods can help to provide data on early life exposure. (2) Methods: Life history calendars were completed by mothers of 8-year-old children from Latinx farmworker and non-farmworker families (*n* = 73 and 65, respectively). Measures were created of months exposure through living adjacent to farm fields and having household members who worked in jobs exposing them to toxic chemicals. Data were divided into time periods of in utero, early childhood (birth-35 months) and later childhood (36–96 months). Cluster analysis compared the measures for children from farmworker and non-farmworker parents. (3) Results: Although, as a group, children from farmworker families have greater lifetime months of probable exposure to pesticides than children in non-farmworker families, cluster analysis reveals groups of children who do not follow that pattern. (4) Conclusions: The life history calendar is a technique for obtaining data on early life toxic chemical exposure that may help assign children to proper exposure groups. Conducting secondary analyses using such information can help to clarify the association of exposures to health outcomes.

## 1. Introduction

Pesticides used in the United States include substances that are potent neurotoxicants. Agricultural workers are exposed directly to multiple classes of pesticides and pesticide residues in their work [1,2,3,4], and these workers are often the vector by which pesticides enter the home and result in indirect exposure of family members [5,6], including pregnant women and children [7]. Family members are also subject to direct exposure by drift from nearby fields and from using pesticides for the control of pests in housing, which is often in poor repair, gardens and yards [8,9].

Children in farmworker families are particularly susceptible to pesticide exposure [10]. Children have greater surface area relative to body volume, so absorb a relatively greater amount of pesticides through the skin than adults [11]. Their metabolic systems are less mature, so their bodies are unable to detoxify some chemicals, such as the organophosphorus pesticides commonly used in agriculture and, thus, they are more vulnerable to their effects than adults [12]. Behaviorally, children come in contact with pesticides through hand-to-mouth activity and playing on contaminated floors [13,14,15]. 

Compared to adults, children are particularly susceptible to effects of early life pesticide exposure. The developing neurological system is characterized by critical periods, with early in utero exposures likely to result in structural malformations, and later in utero and early life exposures expected to result in permanent functional and behavioral alterations [16]. In prospective studies of child cognitive development, children exposed to pesticides prenatally and during early life demonstrate lower IQ by seven years of age [17,18,19]. However, prospective studies are difficult in many vulnerable populations, because of the expense of following children over years, as well as the difficulty of tracking members of a mobile population. Farmworkers, many of whom migrate to follow crops and resist efforts at tracking because of immigration concerns, constitute such a population that is both subject to pesticide exposure, but difficult to follow. Therefore, determining methods of ascertaining prenatal and postnatal exposure without the time, expense and difficulty of a prospective research design is of value.

Life history or life event calendars have been used to aid respondent recall of events occurring long prior to the data collection [20]. This method uses life event cues of significant milestones to encourage respondents to remember less memorable events or circumstances. In environmental health, life history calendars have been used to elicit occupational histories from migrant farmworkers in the US [21], agricultural exposures among minority adult farmers [22], and prenatal and early life pesticide exposures among school children in Nicaragua [23]. In a comparison with a traditional questionnaire method, calendar-based questions were found to be more effective in eliciting the details of complex life histories [24]. In a test-retest assessment of reliability of calendar-based work histories, the calendar was found to be effective, particularly if data were categorized rather than used as direct counts [25].

The goal of this paper is to explore the use of life history calendars, to characterize and compare pesticide exposure in children in Latinx farmworker and non-farmworker families in the US. The specific aims are: (1) to describe the lifetime pesticide exposures of Latinx children whose families either do or do not include farmworkers; (2) to identify the typical profiles of exposure in these children; and (3) to compare the lifetime exposure and typical profiles of exposure in children in farmworker families with those of children in non-farmworker families.

## 2. Materials and Methods 

The study reported here is part of a larger two-group, prospective, longitudinal study, examining the health and cognitive effects of pesticide exposure in children in farmworker families. The larger study uses a comparative design, recruiting a sample of children in families of Latinx farmworkers (the exposed sample) and a sample of children in similar families, but without a member who was a farmworker (the non-exposed sample). 

### 2.1. Inclusion Criteria and Participant Recruitment

Inclusion criteria for the children were similar in both samples. Children were aged 8 years at baseline and had to have completed the first grade in the United States. Children had to be able to cooperate with and complete an assessment battery. All children had to be from families that self-identified as Latino or Hispanic, and with household incomes below 200% of the US federal poverty line. In the farmworker sample, the mother/guardian or her partner must have been employed in farm work on non-organic farms during the past three years. In the non-farmworker sample, adults could not have been employed in industry that involves routine exposure to pesticides (e.g., farm work, landscaping, pest control) in the previous three years. Families in the non-farmworker sample could not have lived adjacent to agricultural fields in the previous three years.

Exclusion criteria for both samples included children having life threatening illnesses, prior history of neurological conditions, physical condition or development disorder that would not allow them to complete or would interfere with the results of neurobehavioral tests or MRIs (used in the larger main study), primary language other than Spanish or English spoken in the home or refusal of mother/guardian to complete the questionnaires.

Children and their mother/guardians were recruited from March 2018, to December 2019. A total of 76 children were recruited for the farm work sample and 66 for the non-farm work sample. Of these, life history calendars were completed by mothers for 73 children in the farm work sample and 65 children in the non-farm work sample. This sample of 138 is used in this paper. For children in farmworker families, the community partner North Carolina Farmworkers Project developed a list of families with an 8-year-old child, and the locations where they lived. This included families who had participated in a previous study of child growth and nutrition. In addition, other community organizations that served farmworker families in the recruitment area were contacted. Study personnel contacted the mothers. Similarly, for the non-farmworker sample, local recruiters in Winston-Salem and community members developed a list. For both samples, mothers were contacted by a bilingual staff member who explained the overall study procedures, answered questions and, if the mother agreed to participate, obtained signed informed consent from the mother and assent from the child. As recruitment progressed, community partners worked with the study team to balance the two samples on socioeconomic status. All procedures were approved by the Wake Forest University Institutional Review Board. The study received a Certificate of Confidentiality from the National Institutes of Health.

### 2.2. Data Collection

The data in this paper were gathered at the baseline visit for the study. Mothers completed a standardized interviewer-administered questionnaire. This questionnaire included demographic and background data on the family and child. This part of the interview was collected using tablets. Data were entered in real time during the interviews using Research Electronic Data Capture (REDCap). REDCap is hosted at Wake Forest School of Medicine through the Clinical and Translational Science Institute. The REDCap system provides secure, web-based applications for a variety of types of research [26].

To collect data on the possible exposures the child might have experienced to pesticides, the mother completed a life history calendar for the child, beginning with prenatal life and ending at the time of the interview. The interviewer used a long paper form, representing the life span of the child, and she wrote information directly onto this form with the mother watching. The mother was queried to identify the child’s birth date (month and year), which was marked on the top row of the calendar with a sticker. Birth month anniversaries up to the eighth birthday were then marked with stickers going forward in the calendar, and 9 months were counted back from the birth month to indicate the approximate date of conception. Other notable family events (e.g., the birth of other children) were added to this first row to help the mother remember context for the child’s life. Data for other rows on the calendar were then queried, in this order: residence (whether the family lived in rural or urban areas, classed as rural if they lived next to fields; type of housing; source of household water), maternal employment (where employed, tasks performed, crops worked if farm work, probing for potential exposures), paternal employment, and employment of other co-resident individuals. Finally, the mother was queried across the calendar for whether pesticides were used within the family dwelling and whether she, the father or any co-resident workers mixed or applied pesticides or other toxic substances. Interviewers were instructed to write notes throughout the calendar form to provide detail that might be important in detecting exposures. 

All interviewers were native Spanish speakers; all spoke English, but with varying degrees of proficiency. Interviewers participated in training, including practice interviews (both questionnaire and life history calendars) that were recorded and critiqued, before any data collection began. Training stressed the need to probe employment of mothers, their spouse or partners, and up to two co-resident adults for interactions with pesticides, cleaning products, paint and other substances that could be neurotoxic. Once data collection began, life history calendars were reviewed upon completion by project staff, and interviewers were asked to re-contact the mother if clarifying information was needed. 

### 2.3. Variable and Measures 

Variables from the baseline questionnaire were used to create measures to describe the sample. These included marital status of the mother (married or living as married versus not married); age at enrollment for the child, mother and spouse/partner; sex of the child; educational attainment of mother and spouse/partner; and current occupation of mother and spouse/partner. 

From the life history calendar interview, the following measures were compiled for the period pregnancy (possible values 1–9 months) and each year of life of the child (1–12 months): months living in housing near agricultural fields, months mother worked in job exposing her to toxic chemicals, months mother’s spouse/partner worked in a job exposing him to toxic chemicals, months up to two other co-resident individuals worked in jobs exposing them to toxic chemicals. Measures were also created for each of these individuals for months worked mixing or applying toxic chemicals. Jobs encountered in the calendars that were considered exposing workers to toxic chemicals included agricultural jobs, including packing and work with livestock, cleaning hotels and restaurants, landscaping, carpet installation and welding. The jobs, including farm work, that were deemed to expose individuals to any form of toxic chemicals are referred to in this paper as “toxic jobs”. Two staff members independently calculated measures from each calendar; their data were compared, and any discrepancies were resolved. The final measures data were entered into a REDcap file.

After these variables were created directly as counts from the calendars, the following cumulative measures were created for three time periods: pregnancy; early childhood, defined as birth month through age 35 months; and later childhood, defined as age 36 months through age 96 months. The measures were: total months the parents (mother plus spouse/partner) worked in toxic jobs; total months the other co-resident adults (up to two) worked in toxic jobs; total months all the adults (mother, spouse/partner, up to two co-resident adults) worked in toxic jobs; total months the parents (mother plus spouse/partner) worked mixing or applying toxic chemicals; total months the other co-resident adults (up to two) worked mixing or applying toxic chemicals; total months all the adults (mother, spouse/partner, up to two co-resident adults) worked mixing or applying toxic chemicals.

No measures were created for home pesticide use. The information provided by the respondents was quite vague. Virtually all respondents in both samples stated that they used pesticides when there were pests in the dwelling, but that use was not routine and not continuous. Therefore, the data appeared to be difficult to quantify or to assign to different life periods.

### 2.4. Data Analysis

Counts/percentages were calculated for data describing the child and mother. We display data for each sample on exposure by month or person-month to assist in visualizing the within and between sample differences. We used negative binomial models to compare the differences in the average number of months of exposures between farmworkers and non-farmworkers. We used a K-means clustering approach to classify children into distinct groups based on the exposures summarized in Section 2.3. The first cluster analysis used the housing and toxic job variables for all three time periods. Pseudo-F statistics and cubic clustering criterion were used to help determine the optimal number of clusters. Similar cluster analysis was performed using the housing and toxic job variables from the in utero and early childhood time periods. All analyses were conducted with SAS 9.4 (Cary, NC, USA).

## 3. Results

### 3.1. Personal Characteristics 

The sample consisted of 69 boys and 69 girls, ages 8 to 9 years of age at recruitment. Most children were born in the US (128, 92.75%). The remainder were born in Mexico (seven, 5.07%) and other Latin American countries (three, 2.17%). Mothers reported almost no use of alcohol (two, 1.45%), tobacco (one, 0.72%), and marijuana or other substances (0%) during the pregnancy. Mothers reported that most of the children were fluent in English (126, 91.30%) and Spanish (108, 78.26%), though only 25 (18.12%) reported that they themselves were fluent in English.

### 3.2. In Utero Exposure

The two samples generally conformed to expectations that more months of in utero pesticide exposure would be reported in the farmworker sample (Table 1). This is clearest for living in housing close to fields, where 60 of 73 farmworker cases lived adjacent to fields for 7 to 9 months of the pregnancy, and 57 of 58 non-farmworker cases never lived adjacent to fields. Farmworker cases reported, on average, 7.42 months living close to fields, compared to 0.16 reported by non-farmworker cases (*p* < 0.001). Comparing the number of person-months household residents worked in toxic jobs, the results were less clear. Nineteen of 73 in the farmworker sample reported 0 person-months, and 16 of the 62 in the non-farmworker sample reported at least seven person-months of work in toxic jobs. Nevertheless, the differences were significant, with farmworker cases reporting an average 8.44 months, compared to 2.32 months in non-farmworker cases, working in toxic jobs (*p* < 0.001). Few of either sample reported household residents mixing or applying chemicals, but more of the farmworker sample reported more than 0 months in these tasks (1.27 months versus 0.52 months, *p* = 0.33).

### 3.3. Early Childhood Exposure

Again, the two samples generally conformed to the expectation that the farmworker sample would have greater exposure than the non-farmworker sample (Table 2). Farmworker cases reported, on average, 31.10 person-months living adjacent to fields, compared to 1.85 person-months among the non-farmworker cases (*p* < 0.0001). More in both samples reported household adults working at least some person-months in toxic jobs than was the case for in utero exposure. However, the farmworker sample had more reports of adults in toxic jobs and had the most extreme reports up to 108 person-months in toxic jobs over the 36 months. For farmworker cases, the mean was 35.18 person-months, versus 12.48 person-months for non-farmworker cases (*p* = 0.0005). Few mothers in either sample reported household residents mixing or applying chemicals (farmworker cases average was 5.42 person-months, compared to 3.02 person-months in non-farmworker cases; *p* = 0.48).

### 3.4. Later Childhood Exposure

As would be expected from the study inclusion criteria, there are clear differences between the samples in later childhood in living adjacent to fields, and in having household members working in toxic jobs (Table 3). Nonetheless, some children in the farmworker sample did not live adjacent to fields and household adults worked limited months in toxic jobs. Similarly, there are children in the non-farmworker sample whose profile looks more like the farmworkers. However, the overall differences were significant. Among the farmworker cases, a mean of 60.68 person-months living adjacent to fields was reported, compared to only 3.32 person-months among the non-farmworker cases (*p* < 0.0001). Similar differences are apparent for adult household residents working in toxic jobs (71.92 person-months, versus 23.43 person-months; *p* < 0.0001). As in the other time periods, few household members in either sample participated in mixing or applying chemicals (14.75 person-months versus 5.95 person-months; *p* = 0.21).

### 3.5. Cluster Analysis

The first cluster analysis using the housing and toxic job variables for all three time periods produced two clusters (Table 4). Cluster 1 included 65 of 73 children in the farmworker sample; cluster 2 included 63 of 65 children in the non-farmworker sample. Examination of the data points for the two non-farmworker children classified into cluster 1 showed that one had lived adjacent to fields consistently through the child’s life, and the other appears to have had exposure due to living near fields and household member toxic jobs in both early and later childhood. For the eight farmworker children who were classified into cluster 2, none lived adjacent to fields in utero and most did not have adults working in toxic jobs in that same period.

The second cluster analysis using data only from the in utero and early childhood time periods also produced two clusters. Cluster 1 included 60 of 73 children in the farmworker sample; cluster 2 included 64 of 65 children in the non-farmworker sample. Examination of the data points for the one non-farmworker child who was classified in cluster 1 showed that the family had lived adjacent to fields consistently through the child’s life. For the 13 farmworker children who were classified into cluster 2, it appears that some did not live adjacent to fields until later childhood, and there was less household member engagement in toxic jobs in early life.

## 4. Discussion

This study used a life history calendar approach to develop indices of exposure of children from time in utero through the first 96 months of life. Using the life history calendar allowed construction of quantitative work and residential histories using proxies for exposure (residence near fields, adult occupational activity) known to be key to the pesticide take-home exposure pathway. We have characterized exposure in three periods: prenatal, early childhood and later childhood. These periods are of value, because the exact critical period for the effects of pesticides on the developing child is not known [27,28]. Furthermore, it is likely that the critical period will vary, depending on the particular class of pesticide, the neurological system in question and the particular outcome examined. The parent study will examine brain anatomy and function, as well as developmental and behavior outcomes, so being able to characterize exposure into at least these three time periods is useful.

Life history calendar studies address the problem of environmental exposures distant in time from their outcomes [21]. While prospective studies would be the optimal means of documenting early life exposures and later outcomes, these are impractical when the population is mobile, when funding is not infinite and when answers to questions concerning the negative consequences of toxic exposures are needed sooner rather than later. Even those important prospective studies of early life pesticide exposure and child outcomes, which have documented the association of pesticide exposure and IQ, have no data before late pregnancy [17,18,19]. Assisting respondents in recalling past events through the use of a calendar helps to provide much needed and targeted early life information. Other methods, like job-exposure matrices, are also difficult to use when the respondent is not necessarily a worker, and when exposures stretch across 9 years, often in two countries, for workers whose tasks are often quite varied, depending on the demands of the employer. 

It is not clear what the best metrics are to describe early life exposure to chemicals such as pesticides. Prior studies have used the single variable proxy of living in communities close to fields for early life pesticide exposure [29] or biomarkers from birth (meconium or umbilical cord blood), which reflect only recent exposures or persistent pesticides [17]. Life history calendars have received only limited attention. This approach was reviewed in a special issue of the American Journal of Industrial Medicine (volume 63, number 4) in 2001, as a means of obtaining prior pesticide exposure data among farmers and farmworkers. Since then, the only reports of the use of such calendars for estimating pesticide exposure are from Costa Rica [30,31] and Nicaragua [23], where they have been used to estimate prenatal and early life exposure of children, as in the current study. In those cases, parents were asked to name specific pesticides to which children were exposed. While knowing the exact pesticide, or at least the class of pesticides, to which a child was exposed could help refine metrics, that is not always possible, and may not even be accurate when provided by the parents. Based on our previous experience with farmworkers in the US, we chose not to focus on specific pesticides, as farmworkers, much less family members who do not participate in farm work, often have no way of knowing the names of pesticides being used.

One expects children in current farm-working families to have a greater lifetime exposure than those in current non-farm working families. From the data collected in this study, that does appear to be the case. Our data show that their lifetime exposure, from time in utero on, is likely high, relative to the children in non-farmworker families. Nevertheless, there is important variability within both groups of children. Within the two earlier time periods, a group of the children in current farmworker families did not live adjacent to fields and had relatively little co-resident adult involvement in toxic jobs. Similarly, there are children in the current non-farmworker families who have a pattern of early exposure similar to children in farmworker families. 

This paper has a number of limitations that should be taken into account. The research was restricted to families in North Carolina. It may be that families elsewhere in the US have different patterns of exposure. While the proportions of children falling (according to the cluster analyses) outside the groups of original assignment is small, those proportions could be quite different in another population. Home use of pesticides was excluded. There is always a risk of fabrication or faulty memory in collecting early life data, and verification is difficult. While one could research vital statistics and other records to find places of birth or dates of immigration, such research could be costly and invade the privacy of these vulnerable participants. Nonetheless, the methods used have been undertaken with success with similar types of populations (e.g., low literacy and low educational attainment) elsewhere. 

## 5. Conclusions

The life history calendar can be used by researchers with Latinx parents in the US to obtain detailed information on factors such as places of residence during pregnancy and the early life of children, which may be key to understanding their exposure to pesticides and other toxic substances. While the method is somewhat time consuming, mothers of children are willing to participate and provide detailed data.

Children classified as coming from current farmworker families include a subset that data from the life history calendar method reveals have had lower than expected early life exposure. Similarly, a subset of children in the families of non-farmworkers have likely had higher exposures than would be expected. Researchers should be aware of this and conduct analyses taking into account these findings. In the case of the parent study, conducting secondary analyses using a cluster assignment, rather than a recruitment assignment, is warranted.

## Figures and Tables

**Table 1:** In utero exposure to pesticides and other toxic chemicals, comparing number and percent for month ranges, by children in farmworker (*n* = 73) and non-farmworker (*n* = 65) families.

	Months					
Variable	Missing	0	1–6	7–9	10–17	18–27
		*n* (%)	*n* (%)	*n* (%)	*n* (%)	*n* (%)
Mother lived adjacent to fields, months						
Farmworker	0	12 (17)	1 (1)	60 (82)	na	na
Non-farmworker	7	57 (98)	0	1 (2)	na	na
Household residents worked in toxic jobs, months						
Farmworker	0	19 (26)	4 (5)	30 (41)	11 (15)	9
Non-farmworker	3	46 (74)	0	15	1	0
Household residents mixed or applied chemicals, months						
Farmworker	0	61 (84)	2 (3)	10 (14)	0	0
Non-farmworker	3	59 (95)	0	2 (3)	1 (2)	0

**Table 2:** Early childhood (birth-35 months) exposure to pesticides and other toxic chemicals, comparing number and non-missing percentages for person-month ranges, by children in farmworker (*n* = 73) and non-farmworker (*n* = 65) families.

	Person-Months						
Variable	Missing	0	1–12	13–36	37–60	61–84	85–108
		*n* (%)	*n* (%)	*n* (%)	*n* (%)	*n* (%)	*n* (%)
Child lived adjacent to fields, person-months							
Farmworker	0	6 (8)	3 (4)	64 (88)	na	na	na
Non-farmworker	4	57 (93)	1 (2)	3 (5)	na	na	na
Household residents worked in toxic jobs, person-months							
Farmworker	0	7 (10)	13 (18)	22 (30)	22 (30)	7 (10)	2 (3)
Non-farmworker	0	39 (60)	4 (6)	18 (28)	3 (5)	1 (2)	0
Household residents mixed or applied chemicals, person-months							
Farmworker	0	56 (78)	6 (8)	11 (15)	0	0	0
Non-farmworker	0	58 (89)	1 (2)	5 (8)	1 (2)	0	0

**Table 3:** Later childhood (36–96 months) exposure to pesticides and other toxic chemicals, comparing number and non-missing percentages for person-month ranges, by children in farmworker (*n* = 73) and non-farmworker (*n* = 65) families.

	Person-Months							
Variable	Missing	0	1–12	13–36	37–60	61–96	97–121	122–204
			*n* (%)	*n* (%)	*n* (%)	*n* (%)	*n* (%)	*n* (%)
Child lived adjacent to fields, person-months (%)								
Farmworker	0	3 (4)	0	2 (3)	4 (5)	64 (88)	na	na
Non-farmworker	3	56 (90)	1 (2)	1 (2)	4 (6)	0	na	na
Household residents worked in toxic jobs, person-months (%)								
Farmworker	0	0	1 (1)	14 (19)	15 (21)	27 (37)	9 (12)	7 (10)
Non-farmworker	0	36 (55)	4 (6)	4 (6)	7 (11)	12 (18)	1 (2)	1 (2)
Household residents mixed or applied chemicals, person- months (%)								
Farmworker	0	49 (67)	2 (3)	8 (11)	1 (1)	13 (18)	0	0
Non-farmworker	0	55 (85)	2 (3)	3 (5)	1 (2)	4 (6)	0	0

**Table 4:** Results of cluster analyses using two variables (months living adjacent to fields and person-months household residents worked in toxic jobs) for three time periods (in utero and early childhood, later childhood) and two time periods (in utero and early childhood), comparing farmworker (*n* = 73) and non-farmworker (*n* = 65) samples. Counts and percentages.

Cluster Analysis Including in Utero, Early Childhood, and Later Childhood Variables			
	Cluster 1 *n* (%)	Cluster 2 *n* (%)	Total
Farmworker	65 (89)	8 (11)	73
Non-farmworker	2 (3)	63 (97)	65
Total	67 (49)	71 (51)	138
**Cluster Analysis Including in Utero, and Early Childhood**			
	Cluster 1	Cluster 2	Total
Farmworker	60 (82)	13 (18)	73
Non-farmworker	1 (2)	64 (98)	65
Total	61 (44)	77 (56)	138
